# Evaluation of immune protection of a multi-antigenic DNA vaccine encoding TgROP6 and TgMIC12 against *Toxoplasma gondii* infection

**DOI:** 10.3389/fvets.2025.1674435

**Published:** 2025-11-14

**Authors:** Bohuai Xu, Yue Bi, Yaowen Wang, Jie Sun, Jia Chen, Jingqi Mu

**Affiliations:** 1 Department of Otorhinolaryngology Head and Neck Surgery, The First Affiliated Hospital of Ningbo University, Ningbo, Zhejiang, China; 2 Ningbo Zhenhai People's Hospital, Ningbo, China; 3 Department of Neurosurgery, Ningbo Key Laboratory of Nervous System and Brain Function, The First Affiliated Hospital of Ningbo University, Ningbo, Zhejiang, China; 4 Health Science Center, Ningbo University, Ningbo, Zhejiang, China

**Keywords:** *Toxoplasma gondii*, *toxoplasmosis*, DNA vaccine, TgROP6, TgMIC12

## Abstract

**Introduction:**

*Toxoplasma gondii* infection causes severe congenital disease and abortion in humans and animals. This study evaluated a novel multivalent DNA vaccine targeting ROP6, and MIC12 for protection against acute (RH strain) and chronic (PRU strain) *toxoplasmosis* in mouse models.

**Methods:**

Eukaryotic plasmids encoding pVAX-ROP6 and pVAX-MIC12 were constructed, and mice were immunized with either single or combined formulations.

**Results and discussion:**

Vaccination elicited a robust Th1-biased immune response, characterized by elevated IgG2a/IgG1 ratios, enhanced cytotoxic T lymphocyte activity, increased CD4+ and CD8+ T cell populations, and elevated production of IFN-γ, IL-12, and IL-2. The dual vaccine demonstrated superior efficacy, significantly prolonging survival following lethal RH challenge (compared to uniform mortality in controls by day 6) and reducing PRU brain cyst burden by 56.6%, outperforming single-gene formulations. Although these results establish pVAX-ROP6/MIC12 as promising vaccine candidates, protection remained partial, highlighting the need for further optimization. Overall, this study underscores the potential of bivalent DNA vaccines to induce broad protective immunity against toxoplasmosis, supporting their continued development for clinical and veterinary use.

## 1 Introduction

*Toxoplasmosis*, caused by the obligate intracellular protozoan *Toxoplasma gondii*, is a globally prevalent zoonosis affecting all warm-blooded vertebrates (1–3). Infection poses significant clinical risks for immunocompromised individuals and developing fetuses (4). In livestock production systems, particularly small ruminants (sheep and goats), *T. gondii* infection induces abortions, resulting in substantial economic losses (5–7). In humans, *T. gondii* causes severe clinical outcomes such as chorioretinitis, lymphadenitis, myocarditis, and polymyositis (8). The symptoms of *T. gondii* infection in adults are mild and includes fever, malaise, and lymphadenitis (8). Congenital *toxoplasmosis* can result in encephalitis, intellectual disability, microcephaly, hydrocephaly, microphthalmia, and jaundice (8). Acute maternal infection can also result in abortion or neonate death (8). *T. gondii* infection in sheep and goats can result in a fetus that is mummified or macerated, fetal embryonic death, stillbirth, and abortion storm, resulting in substantial economic losses. The parasite establishes lifelong persistence in infected hosts through tissue cyst formation, and currently available therapeutics, including pyrimethamine, sulfadiazine, and spiramycin cannot achieve complete parasite eradication (9).

Currently, S48 (Toxovax®) remains the only commercially licensed vaccine against *T. gondii* and has been used to reduce abortion rates in sheep (10). However, as a live-attenuated vaccine, its widespread application is constrained by challenges in manufacturing, including the theoretical risk of virulence reversion (11). These limitations underscore the need for next-generation vaccine platforms that are safer and more practical. While numerous vaccine candidates targeting rhoptry and microneme proteins (including SAG1, ROP5, ROP18, GRA5, GRA7 and MIC4) have demonstrated promise in murine models, none have progressed to widespread application, primarily due to insufficient protection against chronic infection (12–15). There is an urgent need to develop safe and efficacious vaccines capable of preventing *T. gondii* infection in both human and veterinary medicine.

DNA vaccines offer distinct advantages, including simplified manufacturing processes, cost-effectiveness, and the ability to elicit robust immune responses (16). Substantial evidence demonstrates that DNA vaccination against *T. gondii* can simultaneously enhance Th1-type cellular immunity and humoral responses, characterized by elevated proinflammatory cytokine production and parasite-specific antibodies that confer partial protection (17, 18). However, single-antigen vaccines often exhibit limited efficacy. Recent comparative studies reveal that multigenic formulations (e.g., SAG1 + SABP1 or SAG1 + SRS29C) significantly prolong survival duration in murine challenge models with the virulent RH strain, outperforming single-gene vaccines (SAG1, SRS29C, or SABP1 alone) (19, 20). Previous study identified ROP6 and MIC12 as highly immunogenic antigens that remain unexplored for diagnostic or vaccine applications, presenting new opportunities for translational development (21). Also, ROP6 mRNA has been recognized as a promising platform for next-generation *toxoplasmosis* vaccine development (21).

The immunogenic properties of TgROP6 and TgMIC12 position these antigens as promising DNA vaccine candidates capable of conferring robust protection against *T. gondii* infection. ROP6 is a rhoptry protein secreted during the invasion process and contributes to the formation of the parasitophorous vacuole, a key feature of intracellular survival of *T. gondii*. It is expressed predominantly in the tachyzoite stage and has been shown to induce both humoral and cellular immune responses in infected hosts (22). These properties, along with predicted strong T cell epitopes, make ROP6 a compelling vaccine candidate. MIC12 is a microneme protein involved in early host cell attachment, a critical step in *T. gondii* invasion. Given its surface exposure and expression during the invasive stage, it presents a promising target for immune recognition. Furthermore, its conserved sequence and antigenicity in previous proteomic studies support its inclusion as a vaccine antigen (18). Moreover, IL-24 and IL-36γ are promising adjuvants for enhancing protective immunity induced by DNA vaccination against *T. gondii* (23, 24).

This study aimed to: (i) assess the vaccine potential of *T. gondii* virulence proteins TgROP6 and TgMIC12 through construction of recombinant eukaryotic plasmids (pVAX-ROP6 and pVAX-MIC12), and (ii) systematically evaluate the protective efficacy of these DNA vaccines against both acute and chronic *toxoplasmosis* in BALB\c mice. While previous studies have examined TgROP or TgMIC proteins individually, this study is the first to combine TgROP6 and TgMIC12 in a DNA vaccine, leveraging their complementary functions in host cell invasion and parasitophorous vacuole maintenance. This strategy aims to enhance the breadth of the immune response and improve protective efficacy.

## 2 Materials and methods

### 2.1 Mice, parasites and cells

Seven-week-old female BALB\c mice (specific pathogen-free [SPF] grade) were procured from Zhejiang Laboratory Animal Center, Hangzhou (China) and maintained under strict compliance with the Chinese National Laboratory Animal Welfare Guidelines. All experimental procedures were approved by the Institutional Animal Care and Use Committee of the Animal Ethics Committee of Ningbo University (permission: SYXK(ZHE)2019–0005).

For challenge studies, we utilized (i) RH strain (Type I) tachyzoites and (ii) PRU strain (Type II) tissue cysts, both propagated using previously established methods in our laboratory (23, 25). Freshly harvested RH tachyzoites were processed to prepare *Toxoplasma* lysate antigen (TLA) and for total RNA extraction using the RNAprep Pure Tissue Kit (TIANGEN), as previously optimized (26). 293-T cells were maintained in Dulbecco’s modified Eagle’s medium (DMEM; Invitrogen) with 10% heat-inactivated fetal calf serum (FCS), 100 IU/mL streptomycin, and 100 IU/mL penicillin at 37 °C with 5% CO2.

### 2.2 Construction of DNA vaccine plasmid

The coding sequences of TgROP6 and TgMIC12 were amplified from *T. gondii* RH strain tachyzoite cDNA using high-fidelity PCR with the following primer pairs: TgROP6: Forward 5′-GGGGTACCATGCATCCGATATCCTGTT-3′ (*KpnI* site underlined), Reverse 5′-GCTCTAGACTACGCGCGTATCATACG-3′ (*XbaI* site under lined); TgMIC12: Forward 5′-GGGGTACCATGCGTGAATATCCTCTC-3′ (*Kpn*I site underlined), Reverse 5′-GCTCTAGATACCAGTACTAGCAACTT-3′ (*Xba*I site underlined). PCR products were cloned into the pMD18-T vector (Takara Bio, Kusatsu, Shiga, Japan) for bidirectional sequencing, generating pMD-ROP6 and pMD-MIC12. Following sequence verification, ROP6 and MIC12 fragments were excised using KpnI/XbaI (TaKaRa) and subcloned into the eukaryotic expression vector pVAX1. The recombinant plasmids pVAX-ROP6 and pVAX-MIC12 were transformed into *E. coli* DH5α, with positive clones selected through dual restriction analysis and Sanger sequencing. Plasmids were purified using an EndoFree Plasmid Giga Kit (Qiagen Sciences, Germantown MD, USA) and resuspended in sterile PBS with the determination of concentrations of pVAX-ROP6 and pVAX-MIC12 by NanoDrop spectrophotometer at OD260 and OD280 (1 mg/mL, A260/A280 ratio 1.8–2.0). Aliquots were stored at −20 °C until use. For PCR program as follow: Initial Denaturation: 95 °C for 5 min. Amplification Cycles (repeated 30 times). Denaturation: 95 °C for 30 s. Annealing: 60 °C for 30 s. Extension: 72 °C for 1 min. Final Extension: 72 °C for 5 min. Hold: 4 °C forever.

### 2.3 The expression of recombinant plasmid *in vitro*

To confirm recombinant plasmid expression, Human Embryonic Kidney (HEK) 293-T cells transfected with pVAX-ROP6 or pVAX-MIC12 was detected by indirect immunofluorescence assay (IFA). In brief, the recombinant plasmid pVAX-ROP6 or pVAX-MIC12 was transfected into Human Embryonic Kidney (HEK) 293-T cells using LipofectamineTM 2000 (Invitrogen, Carlsbad, CA, USA) following the manufacturer’s protocol. At 48 h post-transfection, cells were fixed with ice-cold acetone for 15 min and permeabilized with PBS containing 0.1% Triton X-100 (PBST). After three washes with PBST, cells were incubated with goat anti-*T. gondii* polyclonal antibody (1:100 dilution in PBST) (Abcam, Cambridge, MA, USA) at 37 °C for 1 h, followed by incubation with FITC-conjugated donkey anti-goat IgG secondary antibody (Proteintech Group Inc., Chicago, IL, USA; 1:100) at room temperature for 45 min. Fluorescence signals were visualized using a Zeiss Axio-plan fluorescence microscope (Carl Zeiss, Oberkochen, Germany). Cells transfected with empty pVAX1 vector served as negative controls.

### 2.4 Immunization and challenge

Experimental groups (*n* = 30 per group) received intramuscular immunizations with 100 μL (100 μg) of DNA vaccines - pVAX-ROP6, pVAX-MIC12, their binary (1:1, the dual vaccine was mixed before injection) combination, − administered at 2-week intervals, while control groups received PBS, empty pVAX1 vector, or remained naive. Serial blood collections at weeks 0, 2, 4, and 6 post-immunization yielded sera through clotting (37 °C, 30 min) followed by centrifugation (4,000 × g, 5 min, 4 °C). Two weeks post-final immunization, parallel challenge studies were conducted: (i) intraperitoneal injection of 1 × 10^3^ RH strain tachyzoites (*n* = 10/group) with 30-day survival monitoring, which is widely used to induce a lethal acute infection in mouse models, enabling clear evaluation of vaccine-induced protection in a stringent model (27, 28) and (ii) oral inoculation with 20 PRU strain cysts (*n* = 5/group) followed by brain cyst burden quantification at 4 weeks post-infection, which was chosen for oral challenge to simulate natural infection and assess the ability of the vaccine to reduce chronic cyst formation in the brain, as supported by earlier studies using comparable models (26, 29).

**Figure 1:**
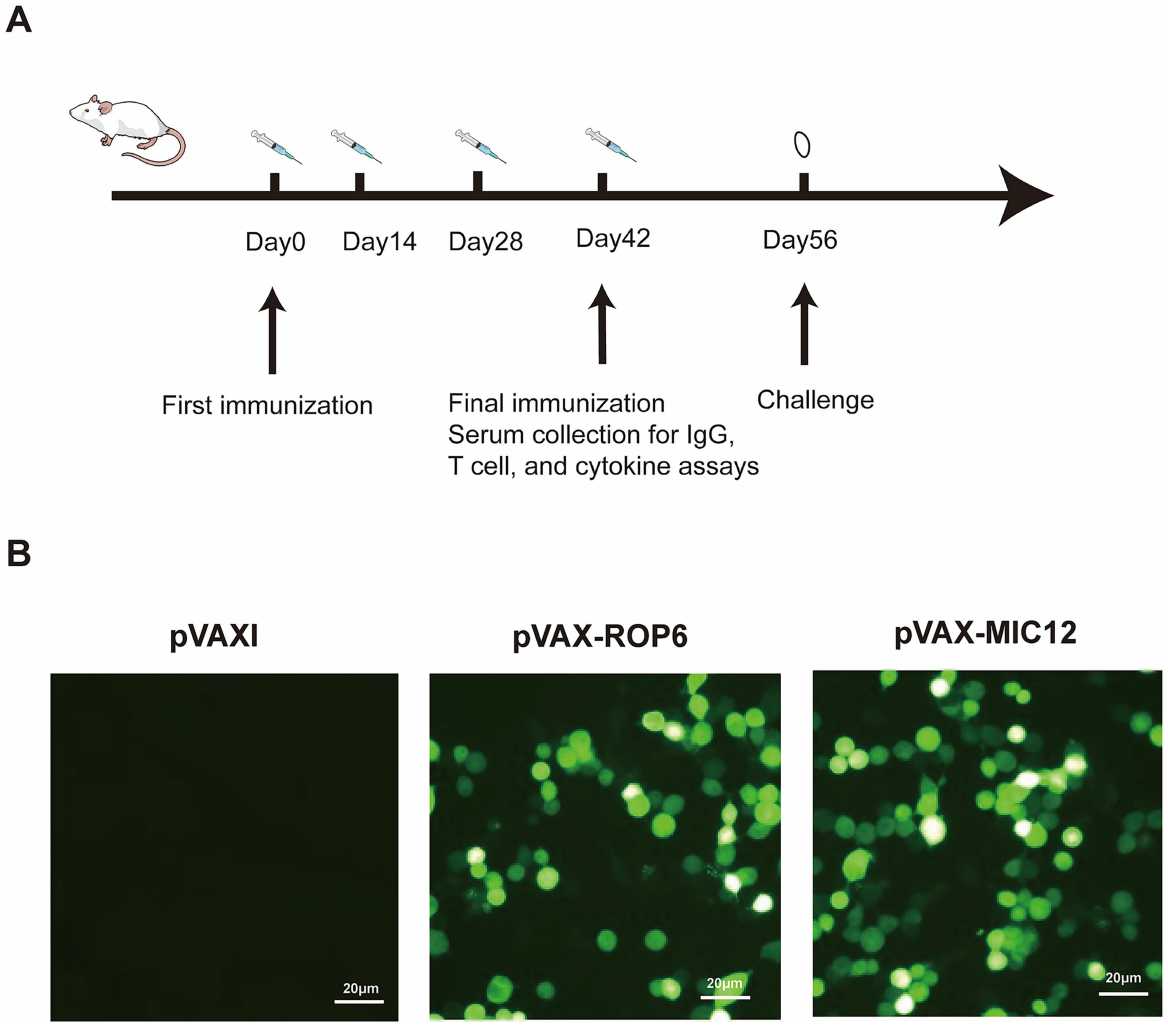
Flow chart of the vaccine immunization strategy and *in vitro* antigen expression validation. **(A)** Flow chart of mice immunization and immunological analyses. **(B)** Protein detection in cells transfected with either empty pVAX I vector (control), pVAX**-**ROP6, or pVAX-MIC12 constructs.

Two weeks after the final immunization, splenocytes were harvested from nine mice per group and allocated for different assays: flow cytometric analysis, lymphoproliferation assays, and cytokine measurements (five mice per assay, with samples pooled as needed), with all measurements performed in triplicate using independent biological replicates. The overall mice immunization and immunological analyses is outlined in the flowchart in Figure 1A. Experimental design is shown in Table 1.

**Table 1:** Experimental design.

Group	Treatment	Immunization	Challenge	Outcome Measured
Group 1	pVAX-ROP6	i.m. ×3, 2-wk interval	RH tachyzoites (i.p.) & PRU (p.o.)	Cyst burden, survival
Group 2	pVAX-MIC12	i.m. ×3	RH & PRU	Cyst burden, survival
Group 3	ROP6 + MIC12	i.m. ×3	RH & PRU	All assays
Group 4	PBS	None	RH & PRU	Control
Group 5	Empty vector	i.m. ×3	RH & PRU	Control
Group 6	Naive	None	RH & PRU	Control

### 2.5 Antibody analysis

Serum levels of anti-*T. gondii* IgG, IgG1, and IgG2a were quantified by ELISA using the SBA Clonotyping System-HRP Kit (Southern Biotech Co., Ltd., Birmingham, UK) at weeks 0, 2, 4, and 6 post-immunization following previously described methods (26). Briefly, 96-well plates were coated with 100 μL/well of TLA (10 μg/mL in PBS) and incubated overnight at 4 °C. After three washes with PBST (PBS + 0.05% Tween-20), plates were blocked with 5% BSA/PBS for 1 h at 37 °C. Serum samples (100 μL/well), diluted in PBS, were incubated for 1 h at room temperature, followed by incubation with HRP-conjugated anti-mouse IgG/IgG1/IgG2a antibodies (1:5,000 dilution) for 1 h at 37 °C. Colorimetric development was achieved using ABTS substrate solution (1.05% citrate buffer [pH 4.0], 1.5% ABTS, 0.03% H2O2; 100 μL/well, 30 min RT), with absorbance measured at 405 nm (BioTek EL × 800, Winooski, VT, USA). All experimental and control samples were run in triplicate.

### 2.6 Lymphocyte proliferation assayed by MTT

Two weeks after the last immunization, splenocytes were collected from three mice of each group as described previously (20). After the erythrocytes were lysed using erythrocyte lysis buffer (0.15 M NH4Cl, 1.0 M KHCO3, 0.1 M EDTA,ph 7.2; Sigma, St. Louis, MO, USA), the splenocytes were resuspended in DMEM medium supplemented with 10% fetal calf serum(FCS). In brief, 3 × 106 cells per well were cultured in 96-well Costar plates and treated with TLA (10 μg/mL), concanavalin A (ConA) (5 μg/mL; Sigma), or medium alone (negative control) at 37 °C under 5% CO2 for 72 h. Thereafter, 10ul of 3-(4, 5-dimethylthylthiazol-2-yl)-2, 5-diphenyltetrazolium bromide (MTT, 5 mg/mL, Sigma) was added to each well, and incubated for 4 h. The proliferative activity was measured using a 3-(4,5-dimethylthiazol-2-yl)-2,5-diphenyl-tetrazolium bromide (MTT, 5 mg/mL, Sigma) dye assay according to the method described by Bounous et al. (30). The proliferative activity was measured using MTT dye assay according to the formula: (OD570 TLA/OD570 Control):(OD570 ConA/OD570 Control). All experimental and control samples were run in triplicate.

### 2.7 Flow cytometry assay

The frequencies of T lymphocyte subsets, including CD4^+^ and CD8^+^ T cells, were analyzed and quantified by flow cytometry as described previously (26, 31). Briefly, single-cell splenocyte suspensions were stained with fluorochrome-conjugated monoclonal antibodies (PE-anti-CD3, APC-anti-CD4, and FITC-anti-CD8; eBioscience, San Diego, CA, USA) for 30 min at 4 °C in the dark. Cells were washed twice with PBS (2 mL) and fixed in FACScan buffer (PBS containing 1% FBS and 0.1% sodium azide) with 2% paraformaldehyde. To quantify cytokine production ex vivo, single-cell suspensions were cultured in RPMI 1640 supplemented with 10% FBS and stimulated for 4 h at 37 °C with 50 ng/mL PMA and 3 μM ionomycin in the presence of 2.5 mg/mL Brefeldin A (Biolegend, San Diego, CA, USA) to enable intracellular cytokine accumulation. Following surface marker staining, cells were fixed and permeabilized for 30 min at 4 °C using BD Cytofix/Cytoperm (BD Biosciences, San Jose, CA, USA), then washed with 1X Permeabilization Buffer (Invitrogen, Carlsbad, CA, USA) prior to intracellular staining with anti-IFN-*γ* (eBioscience, San Diego, CA, USA), anti-Granzyme B (eBioscience, San Diego, CA, USA). Samples were acquired on a BD FACScan flow cytometer (BD Biosciences, San Jose, CA, USA) and analyzed using SYSTEM II software (Coulter, Brea, CA, USA). All experimental and control samples were run in triplicate.

### 2.8 Cytokine assay

Splenocytes were cultured in 96-well plates under antigen stimulation (TLA, 10 μg/mL). Cell-free supernatants were collected at defined timepoints for cytokine profiling: TNF-α at 48 h; IL-2 and IL-4; IL-10 at 72 h; IFN-γ and IL-12 at 96 h. Cytokine concentrations were quantified using commercial ELISA kits (Biolegend, San Diego, CA, USA) with by comparing sample measurements to standard curves generated using mouse recombinant TNF-α, IFN-γ, IL-2, IL-4, IL-12, and IL-10. All experimental and control samples were run in triplicate.

### 2.9 CTL activity assessment

Following spleen lymphocyte isolation, CTL activity was assessed using the CytoTox96® Non-Radioactive Cytotoxicity Assay Kit (Promega, Madison, WI, USA) as previously described (23, 25). Briefly, spleen cells were stimulated with 100 U/mL recombinant murine IL-12 (eBioscience, San Diego, CA, USA) and served as effector cells. Target cells consisted of Sp2/0 mouse cells transfected with eukaryotic expression plasmids (pVAX-ROP6, pVAX-MIC12 or pVAX-ROP6 + pVAX-MIC12) using Lipofectamine™ 2000 reagent (Invitrogen, Carlsbad, CA, USA) following the manufacturer’s protocol. Effector and target cells were co-cultured at ratios of 10:1, 20:1, 40:1, and 80:1 for 6 h. Specific lysis was then calculated using the formula: Specific Lysis (%) = (Experimental − Effector spontaneous − Target spontaneous)/(Target maximum − Target spontaneous) × 100. All experimental and control samples were run in triplicate.

### 2.10 Statistical analysis

Statistical analyses were performed using GraphPad Prism 5.0 (GraphPad Software) and SPSS 17.0 (IBM). Continuous variables (antibody titers, cytokine concentrations) were compared using one-way ANOVA with Bonferroni *post hoc* test for multiple comparisons. Survival curves following RH strain challenge were analyzed by the Kaplan–Meier method with log-rank (Mantel-Cox) testing. All tests were two-tailed, with statistical significance defined as *p* < 0.05. Data are presented as mean ± SEM unless otherwise specified.

## 3 Results

### 3.1 Expression of pVAX-ROP6 and pVAX-MIC12 plasmids *in vitro*

Immunofluorescence analysis revealed distinct intracellular green fluorescence signals in Human Embryonic Kidney (HEK) 293-T cells expressing pVAX-ROP6 or pVAX-MIC12 (Figure 1B), confirming successful recombinant protein expression. In contrast, vector-transfected controls (pVAX I) exhibited no detectable fluorescence (Figure 1B), validating the specificity of the observed signals.

### 3.2 Humoral responses induced by DNA immunization

**Figure 2:**
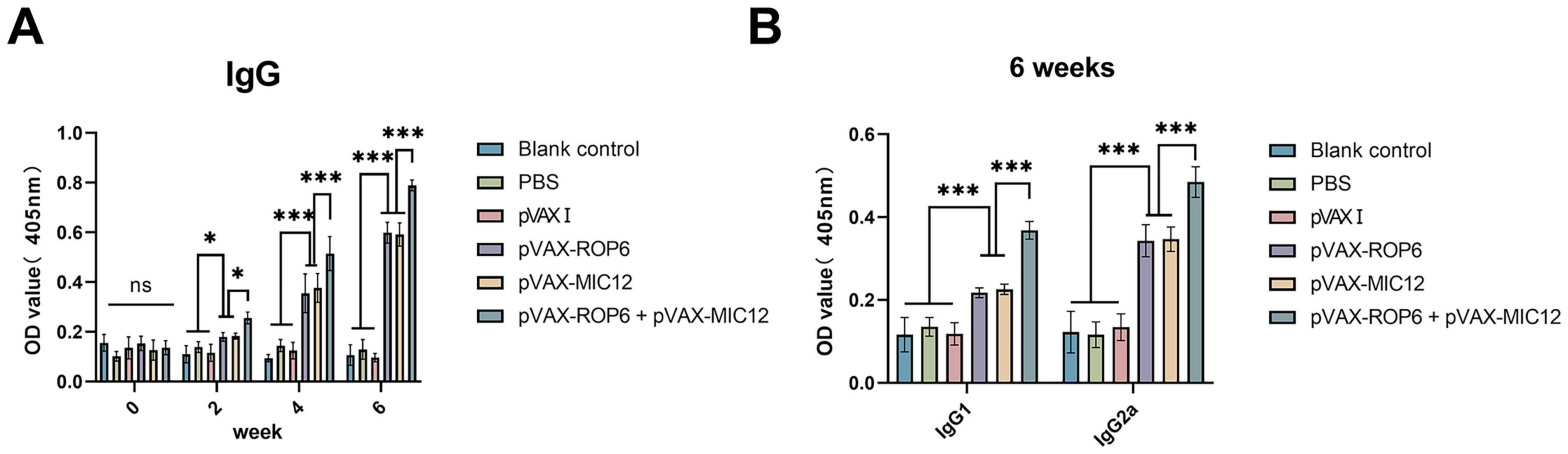
Assessment of humoral immunity induced by single or bivalent gene DNA vaccination. **(A)** Serum anti*-T. gondii* IgG antibody levels in BALB\c mice at weeks 0, 2, 4, and 6 post-immunization. **(B)** Levels of IgG1 and IgG2a subclasses measured 2 weeks after the final immunization. Data are presented as means ± SD (ns, no significant, *p < 0.05, ***p < 0.001).

Serum antibody responses were quantified by ELISA (Figure 2). Mice immunized with single- or double-gene constructs (pVAX-ROP6, pVAX-MIC12, or pVAX-ROP6 + pVAX-MIC12) exhibited significantly elevated total anti-*T. gondii* IgG levels compared to control groups (*p* < 0.05). Also, the increase in antibody levels occurred with successive DNA immunizations (*p* < 0.05). The dual vaccine (pVAX-ROP6 + pVAX-MIC12) induced a higher anti-*T. gondii* IgG production, demonstrating a significantly enhanced effect compared to either treatment alone (Figure 2A). Notably, all vaccinated groups showed a Th1-skewed response, evidenced by elevated anti-*T. gondii* IgG2a/IgG1 ratios (Figure 2B). This bias was higher pronounced in the bivalent group (*p* < 0.01), consistent with robust cellular immunity.

### 3.3 Cellular responses induced by DNA immunization

**Figure 3:**
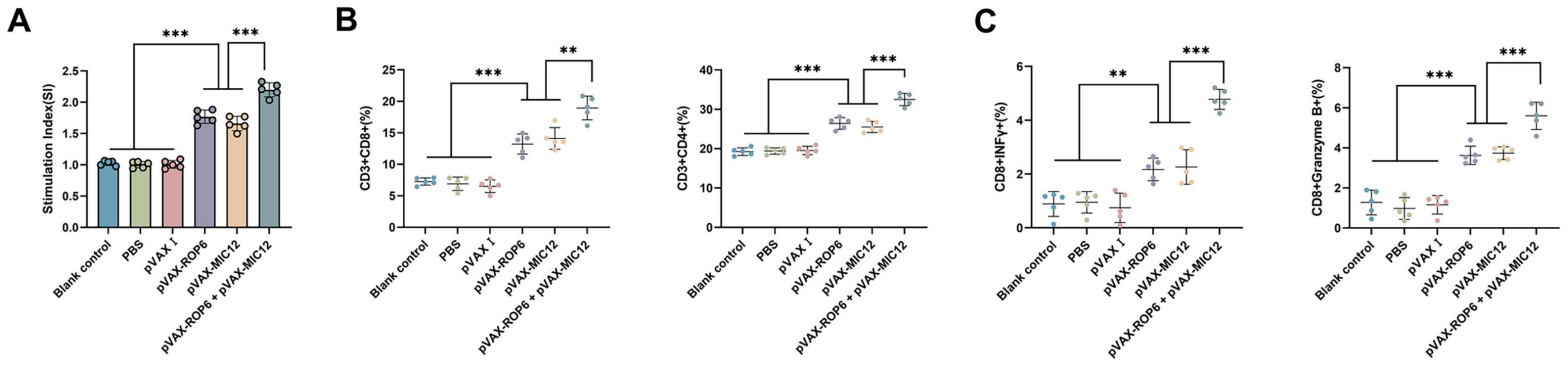
Cellular immune responses induced by single or bivalent gene DNA vaccination. **(A)** Splenocyte proliferation in vaccinated and control groups. **(B)** Frequencies of CD4+ and CD8+ T cells in immunized versus control mice. **(C)** Percentages of IFN-γ and Granzyme B in CD8+ T cells. For flow cytometric analysis, a minimum of 1 × 10^5^ T cells per sample were acquired. Data are presented as mean ± SD (**p < 0.01, ***p < 0.001).

Splenocyte proliferative responses were assessed via MTT assay (Figure 3A). The bivalent vaccine group (pVAX-ROP6 + pVAX-MIC12) exhibited the highest stimulation index (SI) among all groups (*p* < 0.05), demonstrating superior T cell activation. While the mono formulation (pVAX-ROP6 or pVAX-MIC12) also induced significantly higher SI values than control groups, but there were no any significant differences between single-antigen vaccines (*p* > 0.05). Also, no notable proliferation was observed in control groups (*p* > 0.05).

Flow cytometric analysis revealed significant expansion of antigen-specific T cell populations in vaccinated mice (Figure 3B). The bivalent vaccine group (pVAX-ROP6 + pVAX-MIC12) demonstrated the highest CD8 + T cell frequency (*p* < 0.05 vs. all groups), which outperformed single-antigen vaccines (pVAX-ROP6 or pVAX-MIC12) (*p* < 0.05). A parallel trend was observed for CD4+ T cells, with all vaccinated groups showing elevated percentages compared to controls (*p* < 0.05). Similarly, immunophenotyping of CD8 + T cells revealed that the bivalent vaccine robustly induced the highest frequencies of IFN-*γ* and Granzyme B producing cells, significantly outperforming all single-antigen formulations (Figure 3C). No significant differences were detected among control groups (*p* > 0.05).

### 3.4 Measurement of cytokine secretion and cytotoxic T lymphocyte response

**Figure 4:**
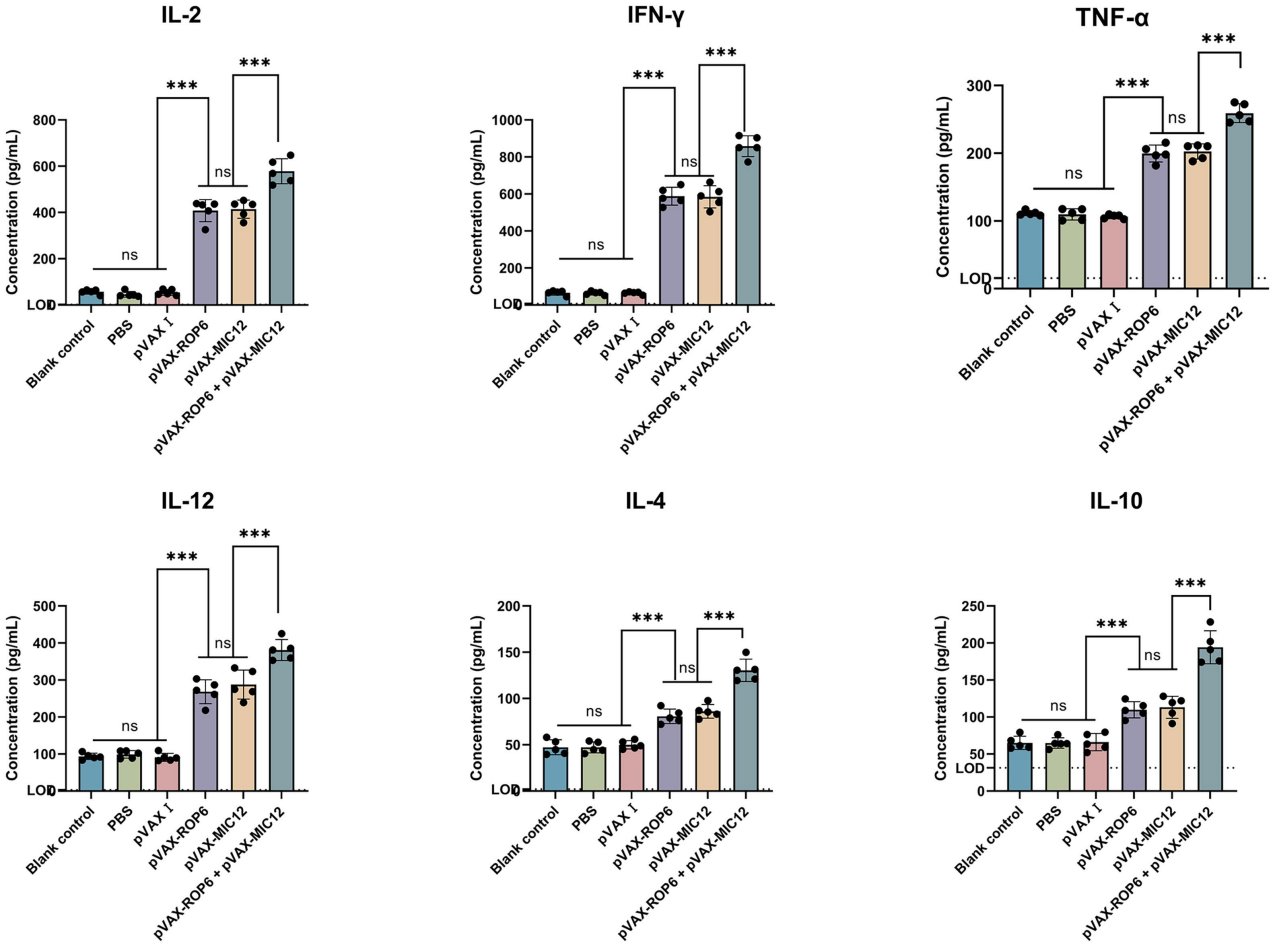
Cytokine secretion profiles of splenocytes from mice immunized with single or bivalent gene DNA vaccines. The dashed horizontal line indicates the lower limit of detection (TNF-α, LOD = 15.6 pg./mL, IFN-γ, LOD = 8 pg./mL, IL-2, LOD = 2 pg./mL, IL-4, LOD = 2 pg./mL, IL-12, LOD = 3.2 pg./mL and IL-10, LOD = 31.3 pg./mL). Data are shown as mean ± SD (ns, no significant, **p < 0.01, ***p < 0.001).

Cytokine analysis of splenocyte supernatants (collected 2 weeks post-immunization) revealed significant Th1 polarization in vaccinated mice (Figure 4). Compared to controls, DNA-immunized groups exhibited elevated TNF-α, IFN-γ, IL-2 and IL-12, with the bivalent formulation (pVAX-ROP6 + pVAX-MIC12) showing superior induction over single-antigen vaccines (*p* < 0.05). While IL-4 and IL-10 levels showed modest increases, these changes were not statistically significant in the controls (*p* > 0.05).

**Figure 5:**
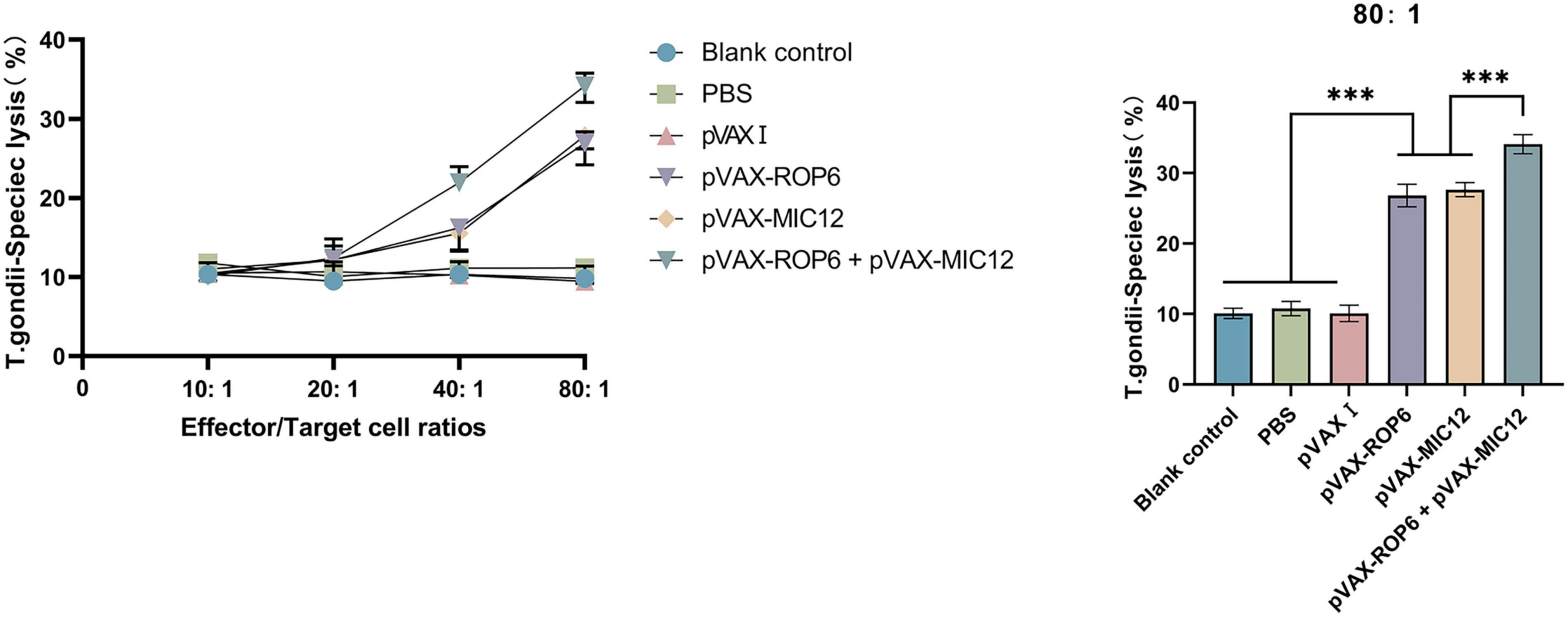
CTL responses of splenic lymphocytes from vaccinated mice. Specific lysis of *T. gondii* infected target cells (Sp2/0 cells transfected with eukaryotic expression plasmids pVAX-ROP6, pVAX-MIC12 or pVAX-ROP6 + pVAX-MIC12) at varying effector to target (E: T) ratios is shown (mean ± SD; ***p < 0.001).

The CTL activity of spleen cells in immunized mice exhibited a dose-dependent enhancement, peaking at an effector-to-target cell ratio of 80:1. Notably, the dual-gene combination (pVAX-ROP6 + pVAX-MIC12) elicited significantly stronger CTL responses compared to single-gene immunizations (Figure 5). In contrast, the three control groups showed comparable CTL activity levels (*p* > 0.05).

### 3.5 Immunoprotection against lethal/nonlethal challenge

**Figure 6:**
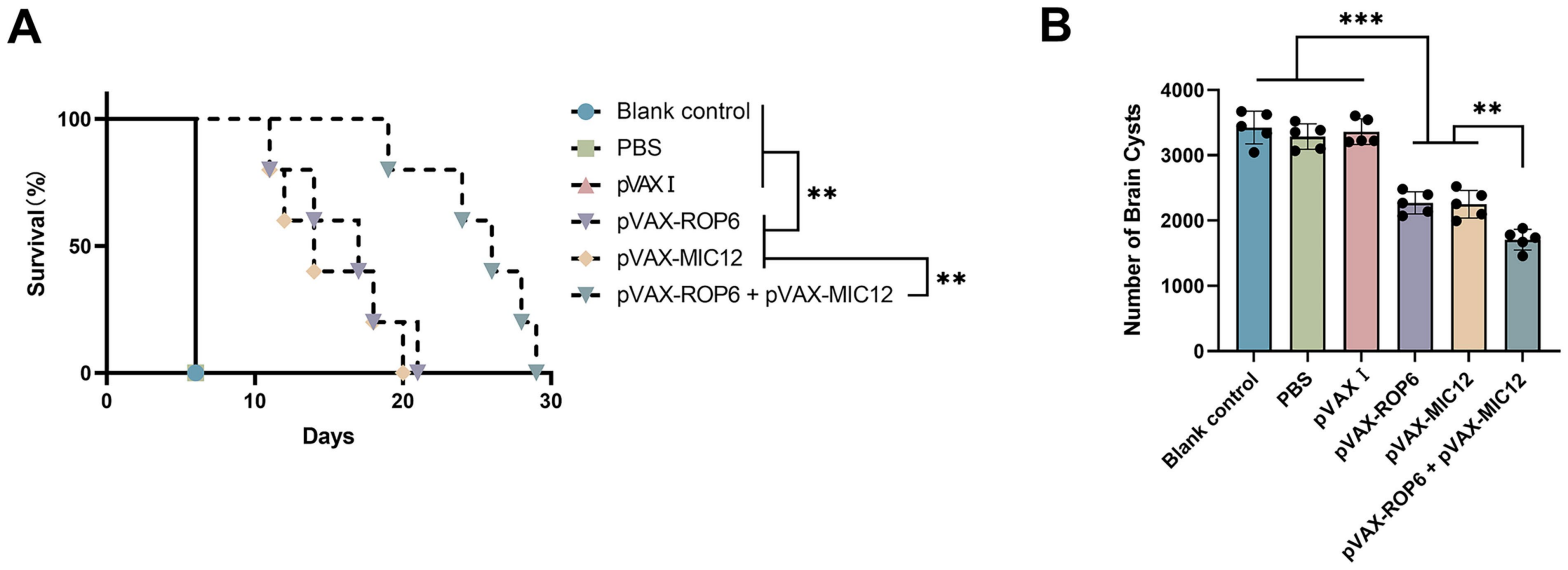
Protective efficacy of vaccination in BALB\c mice. **(A)** Survival rates following intraperitoneal challenge with 1 × 10^3^ RH strain tachyzoites, administered 2 weeks after the final immunization. Survival was monitored over 30 days. Statistical comparison was performed using the log-rank test. The hazard ratio (HR) for the bivalent vaccine group versus the MIC12 group was 0.5385 (95% CI: 0.1559 to 1.860, *p* < 0.01), The HR for the bivalent vaccine group versus the ROP6 group was 0.6538 (95% CI: 0.1893 to 2.259, *p* < 0.01), as determined by Cox proportional hazards regression. **(B)** Brain cyst burden quantified 4 weeks post-challenge with 20 PRU cysts, representing chronic infection. Data are presented mean ± SD (**p < 0.01, ***p < 0.001).

Vaccine efficacy was assessed through two key metrics, including survival after i.p. challenge of tachyzoites of RH strain and cyst burden reduction after oral challenge of cysts of PRU strain (Figure 6). Following i.p. challenge with 1 × 10^3^ tachyzoites of the virulent RH strain, immunized mice exhibited significantly prolonged survival compared to control groups, as depicted in Figure 6A. While control mice succumbed within 6 days post-challenge, DNA immunization using eukaryotic expression plasmids markedly enhanced survival time. No statistically significant differences were observed among the three control groups (*p* > 0.05).

To assess protection against chronic *T. gondii* PRU strain infection, brain cyst burdens were analyzed in immunized and control mice 4 weeks after the final immunization. As illustrated in Figure 6B and Table 2, all vaccinated groups exhibited significantly cyst reduction compared to controls, with the bivalent formulation (pVAX-ROP6 + pVAX-MIC12) demonstrating superior protection (56.6% cyst reduction vs. controls, *p* < 0.01). Cyst burden was also reduced by the DNA immunization with single antigen (35.7% reduction in pVAX-ROP6, or 36.2% reduction in pVAX-MIC12). Control groups showed no statistical differences (*p* > 0.05).

**Table 2:** The number of brain cyst.

Group	Blank control	PBS	pVAXI	pVAX-ROP6	pVAX-MIC12	pVAX-ROP6 + pVAX-MIC12
Mean ± SEM	3,425 ± 112	3,286 ± 87	3,361 ± 88	2,131 ± 42	2091 ± 29	1805 ± 41

## 4 Discussion

DNA vaccines have emerged as a promising immunization strategy against *toxoplasmosis*, demonstrating the capacity to elicit durable humoral and cellular immune responses that confer protection in animal models (25, 26). Among various *T. gondii* antigens investigated, including SAG1, ROP16, MIC4, GRA12 vector-based vaccine, and MIC1/4/13 vaccines have shown particular promise as vaccine candidates (32, 33). While previous studies have indeed investigated members of the TgROP and TgMIC protein families as vaccine candidates, our study is the first to evaluate the combination of TgROP6 and TgMIC12 in a bivalent DNA vaccine platform. Also, TgMIC12 has not previously been used in combination with any ROP antigen, and TgROP6 has only been evaluated individually or in other contexts. This novel antigen pairing was selected based on their complementary roles in parasite invasion and intracellular survival, as well as their high immunogenicity in silico and confirmed expression in both tachyzoite and bradyzoite stages. Our study demonstrates that immunization with either pVAX-ROP6 or pVAX-MIC12 induces robust Th1-polarized cellular immunity, significant humoral responses as well as enhanced protection against both acute (RH strain) and chronic (PRU strain) infection. Notably, the combination with ROP6 and MIC12 exhibited significantly enhanced protective effects, supporting the superior efficacy of multi-antigen approaches observed in other *T. gondii* vaccine studies (17, 19). These findings highlight TgROP6 and TgMIC12 as potent immunogens and reinforce the advantage of combinatorial antigen strategies in *toxoplasmosis* vaccine development (16).

Following *T. gondii* infection, B cell activation leads to the production of parasite-specific antibodies that play a crucial role in host defense (34). These antibodies, particularly IgG, mediate protection by binding to tachyzoite surface antigens, thereby blocking host cell invasion and facilitating macrophage-mediated clearance (25, 34). Consistent with this mechanism, our bivalent antigen vaccine elicited significantly elevated anti-*T. gondii* IgG titers, mirroring the protective humoral responses observed with other antigenic targets like TgROP5 and TgROP18 (14, 35, 36). Notably, our vaccination strategy induced an enhanced IgG1 and IgG2a production and a pronounced IgG2a/IgG1 ratio, dose-dependent increases in antibody titers with bivalent antigen formulations. These findings demonstrate robust Th1 polarization, aligning with established DNA vaccine-induced immune profiles (37, 38). The superior antibody responses generated by the bivalent formulation (ROP6 + MIC12) further support the advantage of multi-antigen approaches in eliciting comprehensive protective immunity.

The adaptive immune response, particularly T cell-mediated immunity, represents the cornerstone of host defense against *T. gondii* infection (37, 39). Our vaccination strategy successfully elicited robust antigen-specific splenocyte proliferation and cytokine production, indicative of protective cellular immunity. The observed Th1-polarized response, characterized by significantly elevated IFN-γ, IL-12, and IL-2 production (40–43), is particularly noteworthy given IFN-γ’s established role in macrophage activation and parasite control during both acute and chronic infection phases (44). The cytokine analysis showed a dominant Th1-type response characterized by elevated IFN-γ and IL-2 levels, with comparatively lower levels of IL-4 and IL-10. While this suggests limited induction of Th2 or regulatory responses, further studies including histopathological evaluation are needed to assess the extent of inflammation or tissue damage. Importantly, the bivalent vaccine formulation demonstrated superior immunogenicity, inducing higher Th1-assosicated cytokine levels along with elevated Th2-associated IL-4 and IL-10 responses compared to single-antigen vaccines, but some previously developed *T. gondii* vaccines, engaging GRA7, ROP21, ROP1 and MYR1 provide only Th1 immune responses without inducing Th2 immunity (45–47). A coordinated Th1/Th2 response is characterized by an optimal IFN-*γ*/IL-10 ratio. This balance reflects the vaccine’s capacity to establish immune homeostasis, a critical determinant of protection against intracellular pathogens (48, 49). The enhanced Th1 response observed with our multi-antigen approach aligns with current understanding of protective immunity while addressing the need for vaccines that elicit comprehensive immune activation against this complex parasite. These findings not only validate TgROP6 and TgMIC12 as potent immunogens but also demonstrate the immunological advantages of multi-antigen formulations in achieving balanced, long-term protection against *toxoplasmosis*.

The coordinated activation of CD4+ and CD8+ T lymphocytes constitutes a critical defense mechanism against *T. gondii* infection, with CD8+ T cells playing a particularly vital role in controlling acute parasitemia through synergistic interactions with CD4+ T cells (50–52). Our findings demonstrate that vaccination with pVAX-ROP6 or pVAX-MIC12 significantly elevated both CD4+ and CD8+ T cell populations compared to controls, with the bivalent formulation (ROP6 + MIC12) showing the most pronounced effect. These results align with previous reports on ROP5/ROP18 and GRA35/42/43 vaccines (25, 36), and suggest that the observed T cell activation may underlie the vaccine’s protective efficacy by: (1) limiting tachyzoite dissemination during acute infection, and (2) reducing cyst formation in chronic stages. The enhanced T cell responses with IFN-γ and Granzyme B elicited by the multi-antigen approach further support the strategic advantage of combinatorial antigen formulations in *toxoplasmosis* vaccine development. Cytotoxic T lymphocytes (CTLs) serve as crucial mediators of immunity against intracellular pathogens, with *T. gondii*-specific CD8 + CTLs demonstrating particular importance in controlling parasitic replication and facilitating pathogen clearance (52, 53). Consequently, eliciting parasite-specific CTL responses represents a cornerstone strategy for developing effective anti-*T. gondii* vaccines. Our findings reveal significantly enhanced CTL activity in splenocytes from vaccinated mice compared to control groups, demonstrating successful induction of pathogen-specific cytotoxic responses. This aligns with recent advances in *T. gondii* vaccinology, including mRNA-LNP (TGGT1_216200) and DNA (GRA24-based) platforms (38, 54), further validating CTL induction as a critical determinant of vaccine efficacy against intracellular parasites.

The genetic and phenotypic diversity of *T. gondii* strains necessitates vaccine candidates capable of eliciting cross-protective immunity. Using the susceptible Kunming mouse model, we demonstrate that vaccination with pVAX-ROP6 or pVAX-MIC12 induces significant protection against both virulent RH (Type I) and avirulent PRU (Type II) strains, confirming the broad protective potential of virulence antigens as seen in prior studies with MIC13/GRA1/ROP7 (14, 32). While robust Th1-polarized responses (characterized by elevated TNF-*α*, IFN-γ, IL-2, IL-12 and IgG2a) and antibody production were observed, protection remained partial, likely due to: (i) incomplete coverage of strain-specific epitopes, (ii) suboptimal T cell (CD4+ T and CD8+ T cells) activation, or (iii) absence of bradyzoite-stage antigens. The superior efficacy of the bivalent (ROP6 + MIC12) vaccine over single-antigen formulations (*p* < 0.05) indicates that combinatorial strategies, which aim to synergistically engage multiple immune pathways, represent a promising approach to enhance protection, as supported by recent hybrid vaccine studies (35, 55). These findings position bivalent antigen vaccines as a promising foundation for developing universally protective *toxoplasmosis* vaccines, though future work should explore incorporation of additional stage-specific antigens to achieve sterile immunity.

One limitation of this study is the lack of serological and histopathological analyses post-challenge, which precludes direct conclusions about humoral or tissue-level immune responses. Although survival benefit was observed, further work is needed to define the immunological mechanisms underlying this protection. In addition, future studies will focus on evaluating specific anti-*T. gondii* IgG responses post-challenge, as well as parasite burden in key tissues (brain, liver, spleen). These analyses will help clarify the immunological basis for the observed protection. Also,initiating a long-term follow-up study in mice to monitor T and B cell memory responses and protection at 3, 6, and 12 months post-vaccination is a critical next step for evaluation of long-term immune memory and durability of protection. Moreover, the critical role of mucosal immunity in defending against *T. gondii* warrants the exploration of mucosal immune analysis, including mucosal IgA and tissue-resident memory T cells in future.

While BALB/c mice are commonly used in preliminary vaccine evaluation due to their consistent immune responses and susceptibility to *T. gondii*, the extent to which these results translate to outbred animals or natural intermediate hosts (e.g., sheep and goats) remains uncertain. Further studies in such models will be essential to confirm the protective efficacy and immune response characteristics of the TgROP6/TgMIC12 vaccine under conditions that more closely mimic natural infection.

In summary, our findings establish TgROP6 and TgMIC12 as promising DNA vaccine candidates capable of eliciting robust humoral and cellular immune responses against both acute and chronic *toxoplasmosis*. The bivalent formulations demonstrated particular efficacy, suggesting significantly enhanced benefits of multi-antigen vaccination strategies. Future development should prioritize: (1) optimization of antigen combinations (e.g., with ROP5/16/18) to enhance protective breadth, and (2) refinement of delivery platforms to maximize immune potency. The vaccine conferred partial protection during acute infection and significantly reduced brain cyst formation following chronic infection. While these findings are promising, further investigation is needed to determine whether this protection extends to distinct parasitic life stages, as this was not directly assessed in the current study. Additionally, we could include a consideration of potential strategies to enhance vaccine efficacy, such as the incorporation of bradyzoite-specific antigens to target the chronic stage more effectively.

## Data Availability

The raw data supporting the conclusions of this article will be made available by the authors, without undue reservation.
