# Supplementary figures and images for: Evaluation of immune protection of a multi-antigenic DNA vaccine encoding TgROP6 and TgMIC12 against Toxoplasma gondii infection
Source: Front Vet Sci. 2025 Nov 14;12:1674435. doi: 10.3389/fvets.2025.1674435 (PMC12662228; doi:10.3389/fvets.2025.1674435)

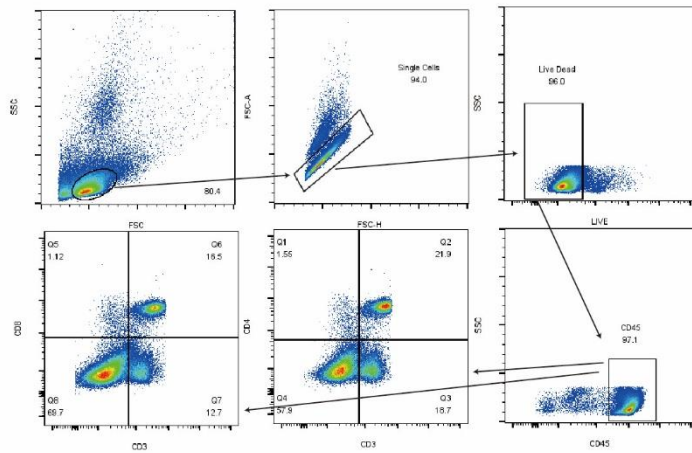

**Supplementary Figure 1.** Flow cytometry gating hierarchy.

Supplement: Supplementary file 1 [file Image_1.pdf]
